# Gene network analysis reveals candidate genes related with the hair follicle development in sheep

**DOI:** 10.1186/s12864-022-08552-2

**Published:** 2022-06-08

**Authors:** Junmin He, Bingru Zhao, Xixia Huang, Xuefeng Fu, Guifen Liu, Yuezhen Tian, Cuiling Wu, Jingyi Mao, Jing Liu, Shuangbao Gun, Kechuan Tian

**Affiliations:** 1 College of Animal Science and Technology, Gansu Agricultural University, Lanzhou, China; 2 College of Animal Science and Technology, China Agricultural University, Beijing, China; 3 College of Animal Science, Xinjiang Agricultural University, Urumqi, China; 4 Key Laboratory of Genetics Breeding and Reproduction of the Fine Wool Sheep & Cashmere Goat in Xinjiang, Institute of Animal Science, Xinjiang Academy of Animal Sciences, Urumqi, China; 5 Institute of Animal Science and Veterinary Medicine, Shandong Academy of Agricultural Sciences, Jinan, China

**Keywords:** Hair follicle, Merino sheep, RNA-seq, Differentially expressed genes, Network

## Abstract

**Background:**

Merino sheep are the most famous fine wool sheep in the world. They have high wool production and excellent wool quality and have attracted worldwide attention. The fleece of the Merino sheep is composed predominantly of wool fibers grown from secondary wool follicles. Therefore, it is necessary to study the development of hair follicles to understand the mechanism of wool production. The hair follicle is a complex biological system involved in a dynamic process governed by gene regulation. The hair follicle development process is very complex and poorly understood. The purpose of our research is to identify candidate genes related to hair follicle development, provide a theoretical molecular breeding basis for the cultivation of fine wool sheep, and provide a reference for the problems of hair loss and alopecia areata that affect human beings.

**Results:**

We analyzed mRNAs data in skin tissues of 18 Merino sheep at four embryonic days (E65, E85, E105 and E135) and two postnatal days (P7 and P30). G1 to G6 represent hair follicles developmental at six stages (i.e. E65 to P30). We identified 7879 differentially expressed genes (DEGs) and 12623 novel DEGs, revealed different expression patterns of these DEGs at six stages of hair follicle development, and demonstrated their complex interactions. DEGs with stage-specific expression were significantly enriched in epidermal differentiation and development, hair follicle development and hair follicle morphogenesis and were enriched in many pathways related to hair follicle development. The key genes (*LAMA5*, *WNT10A*, *KRT25*, *SOSTDC1*, *ZDHHC21*, *FZD1*, *BMP7*, *LRP4*, *T*G*Fβ2*, *TMEM79*, *SOX10*, *ITGB4*, *KRT14*, *ITGA6*, and *GLI2*) affecting hair follicle morphogenesis were identified by network analysis.

**Conclusion:**

This study provides a new reference for the molecular basis of hair follicle development and lays a foundation for further improving sheep hair follicle breeding. Candidate genes related to hair follicular development were found, which provided a theoretical basis for molecular breeding for the culture of fine wool sheep. These results are a valuable resource for biological investigations of fleece evolution in animals.

**Supplementary Information:**

The online version contains supplementary material available at 10.1186/s12864-022-08552-2.

## Background

Subo Merino (SBM) is a superfine wool-producing sheep breed in China. The average wool fiber diameter is 17–19 µm, which surpasses the standard textile count of 80 Nm [1] and has a far-reaching impact on the fine wool sheep industry. The growth and development of sheep wool is controlled by hair follicles (HFs), which are tiny organs attached to the skin that have a complex morphology, complex structure and periodic growth [2]. HFs are composed of multiple cells with very intricate interactions and are involved in the regulation of HF development, growth, regeneration and differentiation. The development of wool follicles has been described in detail for the Merino and it is well established that no new follicles are initiated after birth [3–7]. The first stage in follicle development is the proliferation of epidermal cells to form a placode beneath which an aggregation of dermal cells occurs and the two cell formations grow down together into the dermis. Progressively, the dermal cells move into the epithelial bud to form the pre-papilla and finally the epithelial bulb cells envelop the pre-papilla as the follicle lengthens and descends into the dermis. The stages as they occur in Merino sheep is first follicles formed are the primary follicles (PFs) followed by secondary follicles (SFs) and then secondary-derived follicles (SD) that branch from the SFs [4]. In Merino sheep, fibres from the SD constitute the bulk of the fleece. The first follicles to be initiated in the sheep fetus PFs are visible from 75 days of gestation and are producing a fibre by 90 days of gestation [3]. SFs do not appear until approximately 85 days of gestation. Some of these follicles will begin to branch (SD) at around 105 days [8]. HFs fully mature after birth; therefore, the number of HFs does not increase after birth. In du Cros et al. [9] description of the localization of epidermal growth factor immunoreactivity in sheep skin during wool follicle development, it was found that immunoreactivity was restricted to the periderm and intermediate layers of fetal epidermis at 55 d of gestation, when the first wave of wool follicles are initiated. This particular distribution persisted during subsequent development but never became associated with the basal cells of the epidermis. At approximately 105 d of gestation, however, reactions were detected in the outer root sheath as the follicles matured and in the differentiating cells of the sebaceous glands. Hutchison and Mellor [6] study the effects of maternal nutrition on the initiation of secondary wool follicles in foetal Scottish Blackface sheep. They found initiation of SFs usually takes place between about 95 and 135 days of gestation. Severe underfeeding during the first half of this period did not significantly inhibit the initiation of SFs, but severe underfeeding during the latter half of this period resulted in a significantly lower number of SFs and this number was not increased by refeeding ewes to a high level between 132 days and term. They concluded that SFs initiation is most affected by maternal undernutrition between about 115 and 135 days.

The differentiation of HFs is regulated by a variety of signaling pathways, including the bone morphogenetic protein (BMP), transforming growth factor beta (TGF-β) and Wnt signaling pathways [10, 11]. The specific expression patterns of these molecules in dermal papillae or stromal cells determine their functions during differentiation. However, knowledge regarding the corresponding cellular and molecular mechanisms is limited. After the primary HFs form in the sheep fetus, branches of the primary HFs form secondary HFs [12, 13]. Therefore, it is important to understand the associated molecular gene regulation mechanisms. The wool quality and commercial value of fine wool sheep are determined by the structure and characteristics of their HFs. This branching can be extensive and determines the final follicle population density in which about 80% of the follicles are SD follicles and several wool fibres emerge at the skin surface from the same orifice [14]. To improve the wool yield and wool quality of these sheep, it is necessary to study the factors affecting the formation of HFs and to deeply understand the molecular regulatory mechanisms of HF development. The processes of HF cell development and differentiation are regulated by a variety of genes and multiple signaling pathways; thus, identifying the major genes regulating the development and differentiation of HF cells has become the focus of research.

Wool fiber fineness, fiber length, wool bending, wool strength and hair flexibility determine not only the differences between wool products and other textile fibers but also the craft value of wool textile products [15]. Therefore, to rigorously improve all aspects of animal husbandry, it is important to study the HF development in sheep, and the results have notable practical production value. RNA sequencing (RNA-seq) can identify gene expression differences at the genome-wide level with high reproducibility, accuracy, and reliability and a wide detection range [16]. The practical potential of gene discovery in wool research is the provision of molecular markers for selective breeding and for altering wool growth and wool structure by other biological pathways such as sheep transgenesis that could lead to novel wool properties. In this study, RNA-seq was used to screen genes related to HFs development in order to analyze the expression regulation patterns of these genes at different stages of HFs development in SBM.

## Results

### Overview of sheep skin transcriptomic data

The 18 RNA-seq libraries from skin tissues at six developmental stages generated an average of 12 Gb paired-end clean reads with Q20 values > 95% (Additional file 1: Table S1). In the current study, an average of 84.98% of clean reads from the 18 samples were mapped in pairs. Furthermore, the reads were primarily aligned in gene regions, coding regions, and intergenic regions.

### Analysis of genes associated with sheep skin

**Fig. 1:**
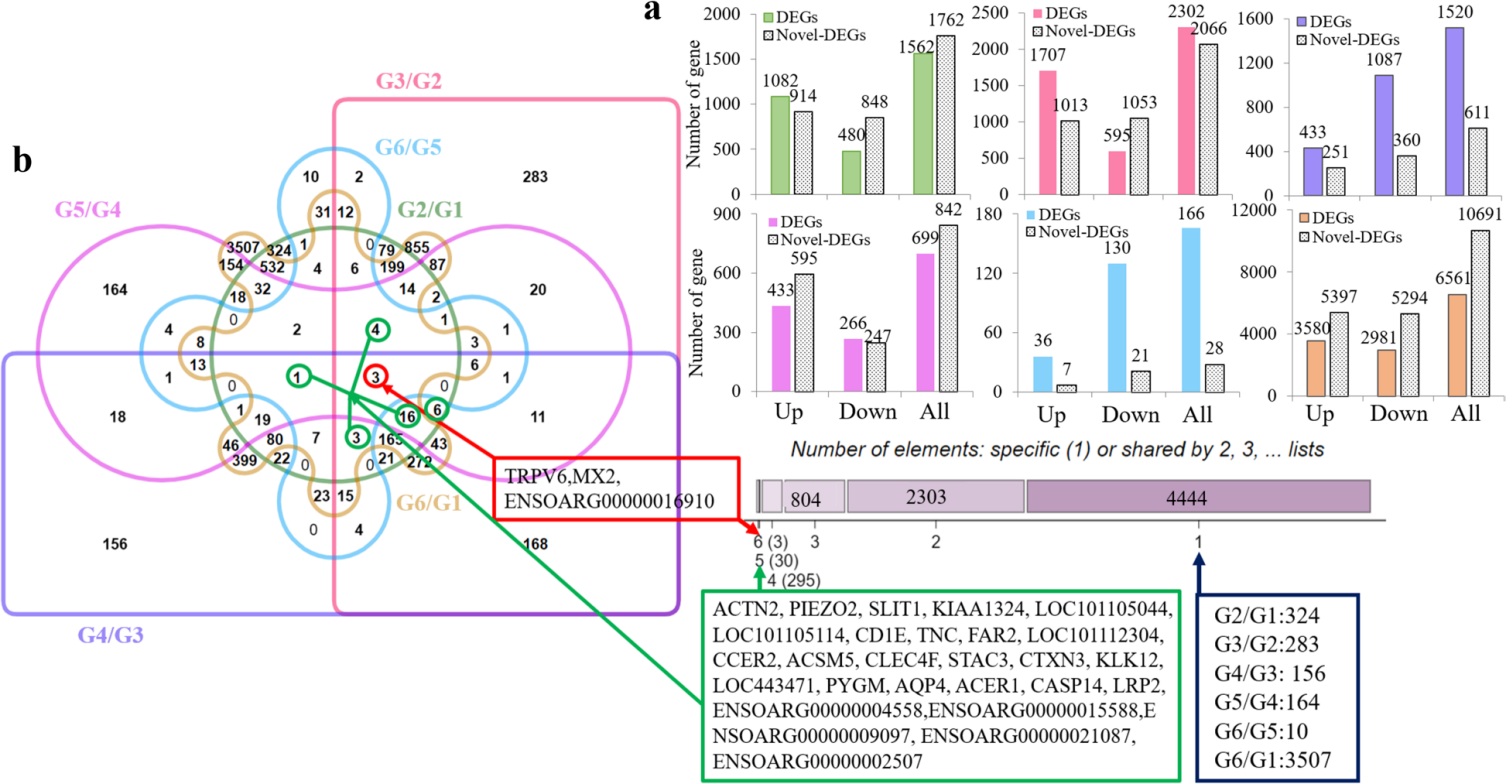
**a** Histogram showing the numbers of DEGs and novel DEGs during HF morphogenesis. **b** Venn diagram of DEGs during HF morphogenesis

A total of 7879 DEGs and 12623 novel DEGs were identified in the six HF development stages of SBM sheep. A total of 1562 DEGs and 1762 novel DEGs were identified in G2/G1, of which 1082 DEGs were upregulated and 480 were downregulated, while 914 novel DEGs were upregulated and 848 were downregulated (Fig. 1a). When G3 was compared to G2, 2302 DEGs (including 1707 upregulated and 595 downregulated DEGs) and 2066 novel DEGs (including 1013 upregulated and 1053 downregulated novel DEGs) were detected. In total, 1520 DEGs (433 upregulated and 1087 downregulated) and 611 novel DEGs (251 upregulated and 360 downregulated) were found between G4 and G3. Between G5 and G4, 699 DEGs (433 upregulated and 266 downregulated) and 842 novel DEGs (595 upregulated and 247 downregulated) were identified. When G6 was compared to G5, 166 DEGs (36 upregulated and 130 downregulated) and 28 novel DEGs (7 upregulated and 360 downregulated) were identified. G6/G1 had the most DEGs, with 3507, and G6/G5 had the fewest DEGs, with only 10. This result indicated considerable differences between the beginning of HF development and postnatal development. The expression of DEGs is shown in heatmaps in Additional file 2: Fig. S1. Venn diagram analysis showed that *TRPV6*, *MX2* and *ENSOARG0000001910* were differentially expressed in all six groups (Fig. 1b).

### DEG enrichment analysis

To further explore the biological functions of DEGs related to HF development in SBM sheep during the fetal period, Gene Ontology (GO) functional analysis and Kyoto Encyclopedia of Genes and Genomes (KEGG) pathway analysis were performed on all 7879 DEGs. In the biological processes (BP) category, the DEGs were mainly associated with the term extracellular fibril organization, cardiac muscle fiber development, skeletal muscle tissue growth, regulation of microvillus assembly and positive regulation of the Wnt signaling pathway (Additional file 3: Fig. S2). In the cellular component category, the DEGs were mainly associated with the terms spectrin-associated cytoskeleton and elastic fiber. In the molecular function category, the DEGs were mainly associated with the term myosin heavy chain binding and monoamine transmembrane transporter activity (Additional file 3: Fig. S2a). It is presumed that the initiation of HF development is mainly driven by the above factors. The significant pathways were mainly concentrated in the cell adhesion molecule (CAM), complement and coagulation cascade, extracellular matrix (ECM) − receptor interaction, hypertrophic cardiomyopathy (HCM), hematopoietic cell lineage, and rheumatoid arthritis pathways (Additional file 3: Fig. S2b).

**Fig. 2:**
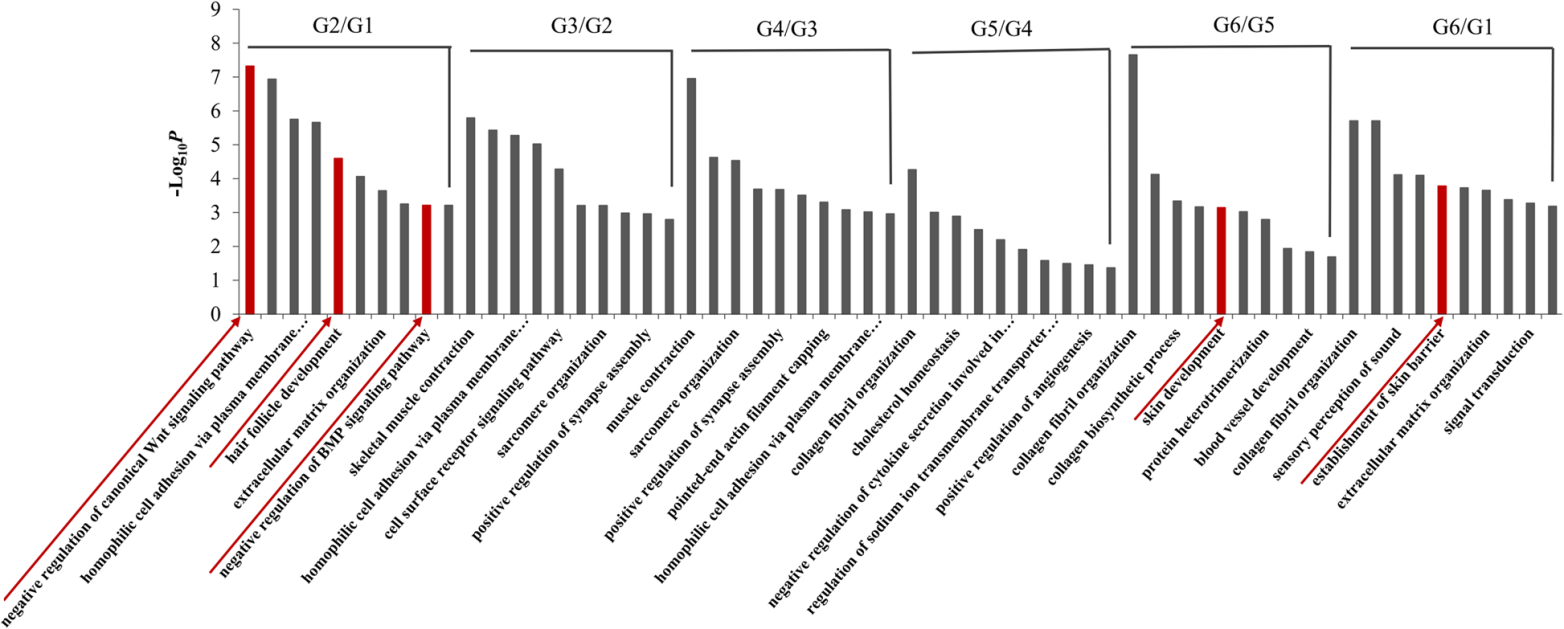
Top 10 GO terms (BP category) during HF morphogenesis

To further elucidate the functions of the DEGs, we also examined the top 10 GO functional enrichment BP terms and KEGG pathways of DEGs in adjacent comparison groups. For G2/G1, the GO terms were enriched mainly in the negative regulation of the canonical Wnt signaling pathway, HF development, and the negative regulation of the BMP signaling pathway, which are associated with HF or skin development (Fig. 2, Additional file 4: Table S2). The GO terms of G3/G2 were enriched in skeletal muscle contraction and muscle contraction. For G4/G3, the terms were mainly concentrated in heart and muscle development. For G5/G4, the terms were mainly enriched in BPs related to signal transduction and the immune response. For G6/G5, the terms were mainly enriched in collagen fiber organization and skin development. In addition, in the comparative analysis between 30 days after birth (G6) and the initial stage of HF development (G1), the DEGs were enriched in processes related to HF development, such as collagen fiber organization and establishment of the skin barrier. In summary, the DEGs in G2/G1, G6/G5 and G6/G1 were directly related to HF development, especially G2/G1. The DEGs in G3/G2 and G4/G3 were related to muscle development, and those in G5/G4 were related to immunity.

**Fig. 3:**
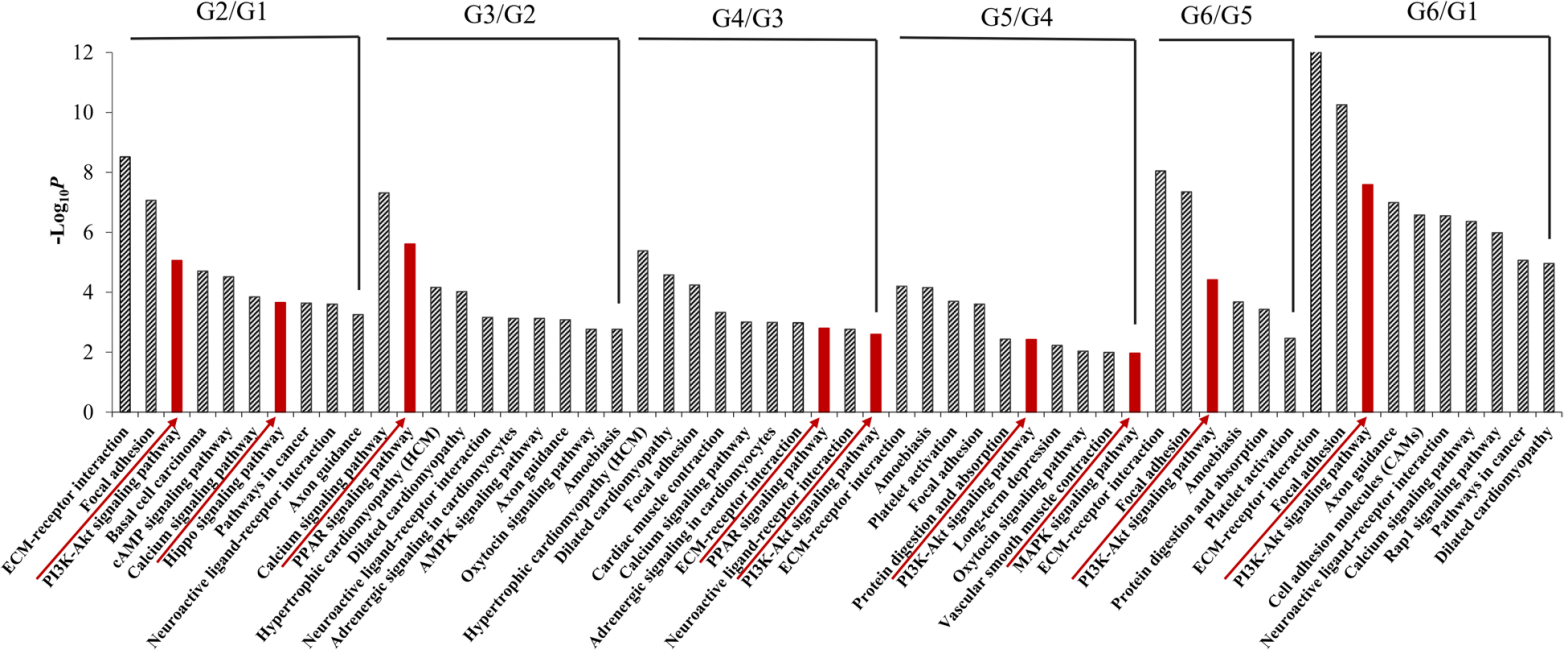
Top 10 enriched KEGG pathways during HF morphogenesis

KEGG enrichment analysis was also performed (Fig. 3, Additional file 5: Table S3). The phosphatidylinositol-3 kinase (PI3K)-Akt signaling pathway (e.g., G2/G1, G4/G3, G5/G4, G6/G5, and G6/G1), Hippo signaling pathway (e.g., G2/G1), peroxisome proliferator-activated receptor (PPAR) signaling pathway (e.g., G3/G2 and G4/G3), mitogen-activated protein kinase (MAPK) signaling pathway (e.g., G5/G4), and ECM–receptor interaction pathways (e.g., G2/G1, G5/G4, G6/G5, and G6/G1), which have been reported to play indispensable roles during HF development, were enriched. We also found that the Hippo signaling pathway was closely related to the development of primary and secondary HFs, and the PPAR signaling pathway and MAPK signaling pathways were related to the stimulation of acquired HFs and the HF cycle.

**Fig. 4:**
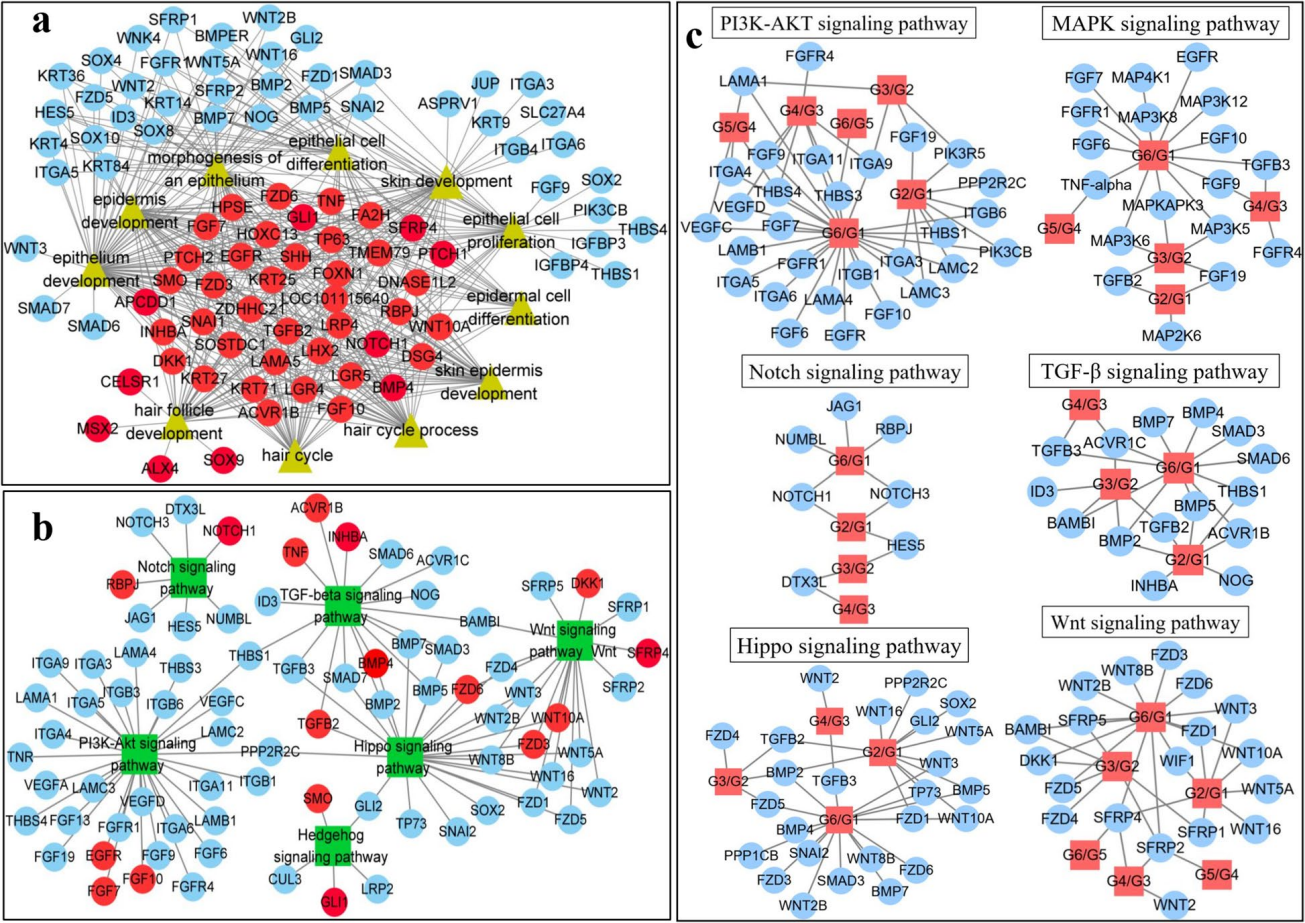
**a** Gene network map of GO terms during HF morphogenesis. **b** Gene network map of KEGG pathways during HF morphogenesis. **c** DEGs in the PI3K-AKT, MAPK, Notch, TGF-β, Hippo, and Wnt signaling pathways during HF morphogenesis

To explore the potential most key genes related to HF morphogenesis, we used Cytoscape to visualize the GO terms related to HF and skin development and the DEGs in KEGG pathways. We found that the enriched BP terms included HF development, the hair cycle process, the hair cycle, skin epidermis development, epidermis development, epidermal cell differentiation, skin development, epithelial cell differentiation, epithelial cell proliferation, epithelium development, and epithelium morphogenesis. The related genes included 41 genes (*ACVR1B*, *APCDD1*, *BMP4*, *DKK1*, *DNASE1L2*, *DSG4*, *EGFR*, *FA2H*, *FGF10*, *FGF7*, *FOXN1*, *FZD3*, *FZD6*, *GLI1*, *HOXC13*, *HPSE*, *INHBA*, *KRT25*, *KRT27*, *KRT71*, *LAMA5*, *LGR4*, *LGR5*, *LHX2*, *LOC101115640*, *LRP4*, *NOTCH1*, *PTCH1*, *PTCH2*, *RBPJ*, *SFRP4*, *SHH*, *SMO*, *SNAIL*, *SOSTDC1*, *TGFβ2*, *TMEM79*, *TNF*, *TP63*, *WNT10A*, and *ZDHHC21*) (Fig. 4a). These genes have certain relationships with the development of HFs, the skin, the epidermis and the epithelium. Similarly, in the pathway analysis, it was found that 17 of the above genes (*ACVR1B*, *BMP4*, *DKK1*, *EGFR*, *FGF10*, *FGF7*, *FZD3*, *FZD6*, *GLI1*, *INHBA*, *NOTCH1*, *RBPJ*, *SFRP4*, *SMO*, *TGFβ2*, *TNF*, and *WNT10A*) were enriched in the TGF-β signaling pathway, Wnt signaling pathway, Notch signaling pathway, Hippo signaling pathway, MAPK signaling pathway, VEGF signaling pathway and Hedgehog signaling pathway, and these pathways are known to be related to skin or HF development (Fig. 4b). Interestingly, in addition to the genes associated with the GO terms and KEGG pathways, many other genes were related to traits in which we were interested (such as *WNT2*, *WNT3*, *TGFβ3*, *KRT14*, *BMP2*, *BMP5*, *BMP7*, *BMPER*, *WNT16*, *WNT5A*, *SFRP2*, *SFRP5*, *SAMD3*, *FGF19*, *FZD1*, and *FZD5*). Similar to the above GO and KEGG analyses, we also carried out KEGG analysis of the pathways related to HF morphogenesis at different stages (Fig. 4c, Additional file 6: Fig. S3a-f) and visualized the genes in the pathways according to the different stages. We found that the results were similar to the above results and identified many genes related to HF morphogenesis.

### K-means analysis of DEGs

**Fig. 5:**
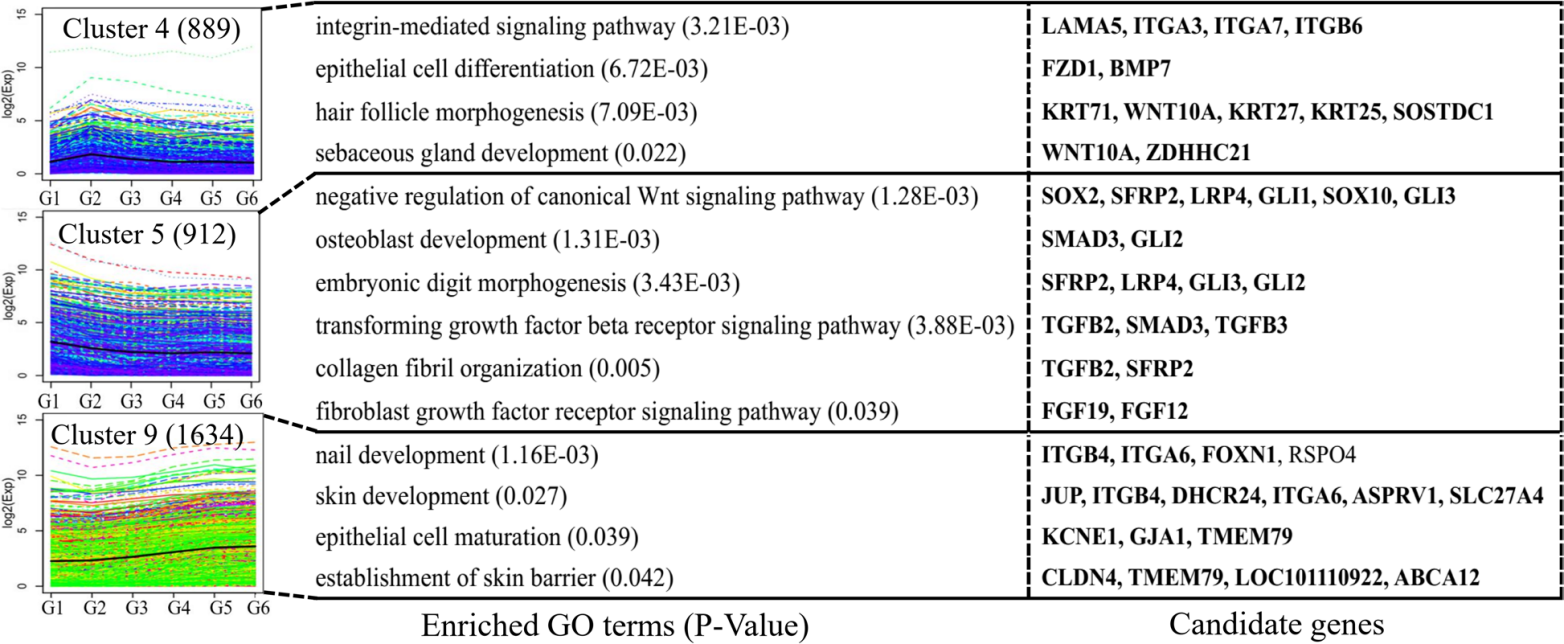
Selected K-means clusters of DEGs corresponding to biological processes, with candidate genes shown next to each cluster. Each box is a differential mRNA cluster, each line in the box represents the expression trend of a differential mRNA, and the black bold line is the average trend of all differential mRNAs in the box

To identify the expression patterns of differentially expressed mRNAs during the six HF developmental stages, we performed k-means clustering of all DEGs and classified them into 10 clusters based on their expression changes (Fig. 5, Additional file 7: Fig. S4). GO analysis (see Additional file 8: Table S4) was carried out on the 10 clusters, and many of the identified BPs were related to the tissue types in which the genes were expressed. In cluster 4, we found that genes were enriched in epithelial cell differentiation (*FZD1* and *BMP7*), HF morphogenesis (*KRT71*, *WNT10A*, *KRT27*, *KRT25* and *SOSTDC1*) and sebaceous gland development (*WNT10A* and *ZDHHC21*), and their expression levels peaked in the G2 period. We also found that the genes in cluster 5 were related to BPs associated with hair fiber or capsule development in the negative regulation of the canonical Wnt signaling pathway (*SOX2*, *SFRP2*, *LRP4*, *GLI1*, *SOX10*, and *GLI3*), embryonic digit morphogenesis (*SFRP2*, *LRP4*, *GLI2*, and *GLI3*), the TGF-β receptor signaling pathway (*TGFβ2*, *SMAD3* and *TGFβ3*), collagen fiber organization (*TGFβ2* and *SFRP2*), cilium assembly and the fibroblast growth factor receptor signaling pathway (*FGF19* and *FGF12*). Surprisingly, the genes in cluster 9 play key roles in skin morphogenesis (*JUP*, *ITGB4*, *DHCR24*, *ITGA6*, *ASPRV1*, and *SLC27A4*), establishment of skin barrier (*CLDN4*, *TMEM79*, *LOC101110922*, and *ABCA12*), and epithelial cell maturation (*KCNE1*, *GJA1*, and *TMEM79*).

### Weighted gene coexpression network analysis (WGCNA)

**Fig. 6:**
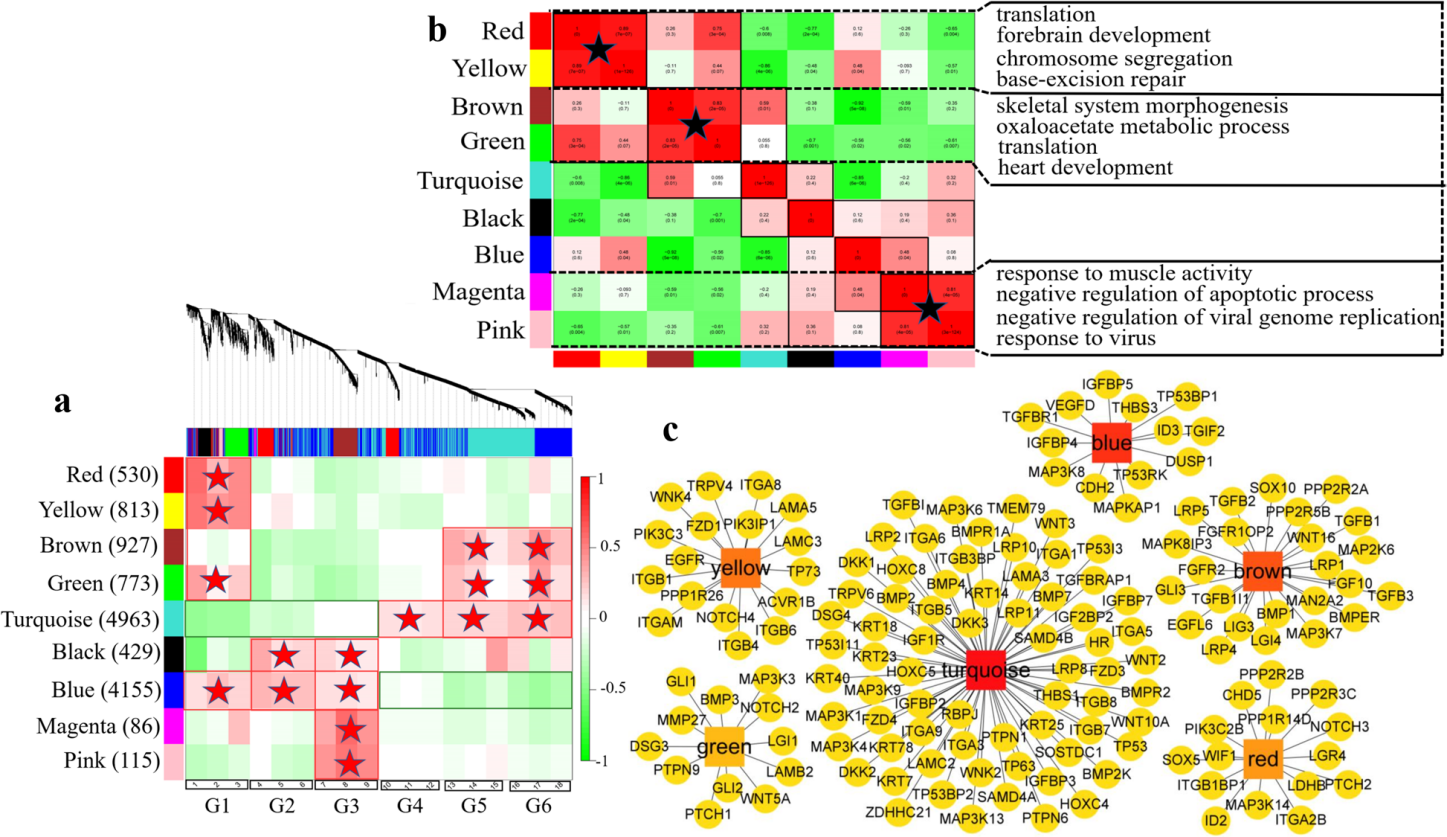
Results of WGCNA. **a** Division of gene modules and the correlations between the gene modules (y-axis) and the sample information (x-axis). The figure shows the clustering of genes, and the division of gene modules was based on this result. Branches of the same color were divided into the same gene modules. **b** Correlations between the gene modules and the module information and GO enrichment (top 3) analysis of the modules. In the panel, a darker color indicates a higher correlation, with red representing a positive correlation and green representing a negative correlation. **c** Visualization of the 150 hub genes related to HF morphogenesis in the modules. A darker color between genes indicates a higher correlation between the genes

We constructed a gene expression matrix consisting of 12791 DEGs from the standardized data (Fig. 6a). The sample expression pattern heatmap showed that genes in the blue module were the most highly expressed at G1, G2 and G3. Those in the turquoise module were most highly expressed from G4 to G6. The similarity between the red and yellow modules was very high (Fig. 6b), and the expression was the highest at G1. Thus, the blue, turquoise, red and yellow modules were selected for further study. GO enrichment analysis was performed for genes in these modules. The 3 most significant terms in the BP category are shown in the figure. Different terms were enriched in each of the nine modules (see Additional file 9: Table S5). The turquoise module contained the most genes (4963), which were involved in negative regulation of cell proliferation, Ras protein signal transduction (GO:0,007,265), and positive regulation of cell migration (GO:0,030,335). The genes in the blue module were mainly associated with the term protein K48-linked ubiquitination (GO:0,070,936), cellular copper ion homeostasis (GO:0,006,878), and glycogen metabolic process (GO:0,005,977). The genes in the brown module were similar to those in the green module and were enriched in skeletal system morphogenesis (GO:0,048,705), face morphogenesis (GO:0,060,325), and heart development (GO:0,007,507). The yellow- and red-module genes were involved in chromosome segregation (GO:0,007,059) and translation (GO:0,006,412). KEGG analysis of the genes in these modules showed that the pathways with significant enrichment included gluconeogenesis, the insulin signaling pathway, the MAPK signaling pathway, the TGF-β signaling pathway, the PI3K-Akt signaling pathway, and other important pathways (see Additional file  10: Table S6). Based on the candidate genes associated with HF development, we found that in the yellow, green, red, brown and turquoise modules (Fig. 6c), the core genes (*LAMA5*, *ACVR1B*, *EGFR*, *FZD1*, *ITFB4*, *PTCH1*, *WNT5A*, *GLI2*, *LGR4*, *PTCH2*, *TGFβ2*, *LRP4*, *LRP5*, *FGF10*, *TGFβ3*, *BMPER*, *SOX10*, *WNT16*, *DKK1*, *TMEM79*, *BMP4*, *DSG4*, *ZDHHC21*, *SOSTDC1*, *TP63*, *RBPJ*, *FZD3*, *KRT25*, *WNT10A*, *WNT3*, *BMP7*, *KRT14*, *FZD4*, and *ITGA6*) may be involved in the control of the HF development process.

### Protein–protein interaction (PPI) network

**Fig. 7:**
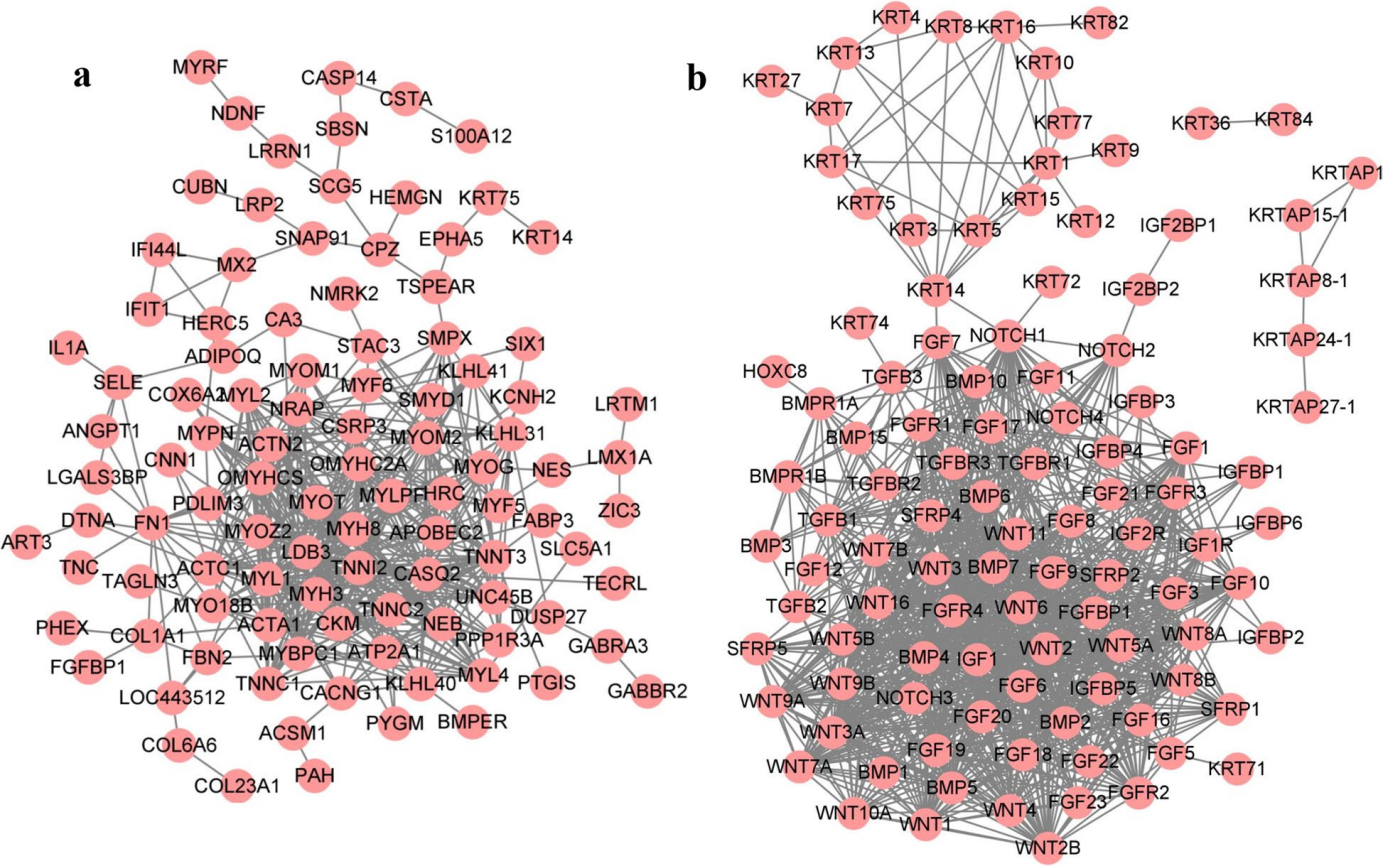
**a** PPI network of the 200 hub DEGs during HF morphogenesis. **b** PPI network of KRT, WNT, FGF, IGF, NOTCH, BMP, TGF, and HOXC family genes

We performed PPI analysis on 200 hub DEGs (Fig. 7a). In the analysis, However, BMPER, FGFBP1, KRT75 and KRT14, which we know may be related to HFs or hair, were prominent. We next determined how the proteins interacted according to gene families related to HF development; thus, we identified KRT, WNT, FGF, IGF, NOTCH, BMP, TGF, and HOXC family genes for PPI analysis (Fig. 7b). We found that these genes constituted 3 interaction networks, among which KRT36 and KRT84 interacted independently, and 5 KRTAP members (KRTAP27-1, KRTAP24-1, KRTAP8-1, KRTAP15-1, and KRTAP11-1) interacted with each other. Most of the proteins had strong interactions. Interestingly, we found that KRT14 associates 17 KRT proteins with other proteins through NOTCH1 and FGF7 and that KRT14, NOTCH1 and FGF7 interact with each other, indicating that these three proteins play key roles in this interaction network. The above description KRT14 will become the focus of our attention.

### Validation of the RNA-seq data

**Fig. 8:**
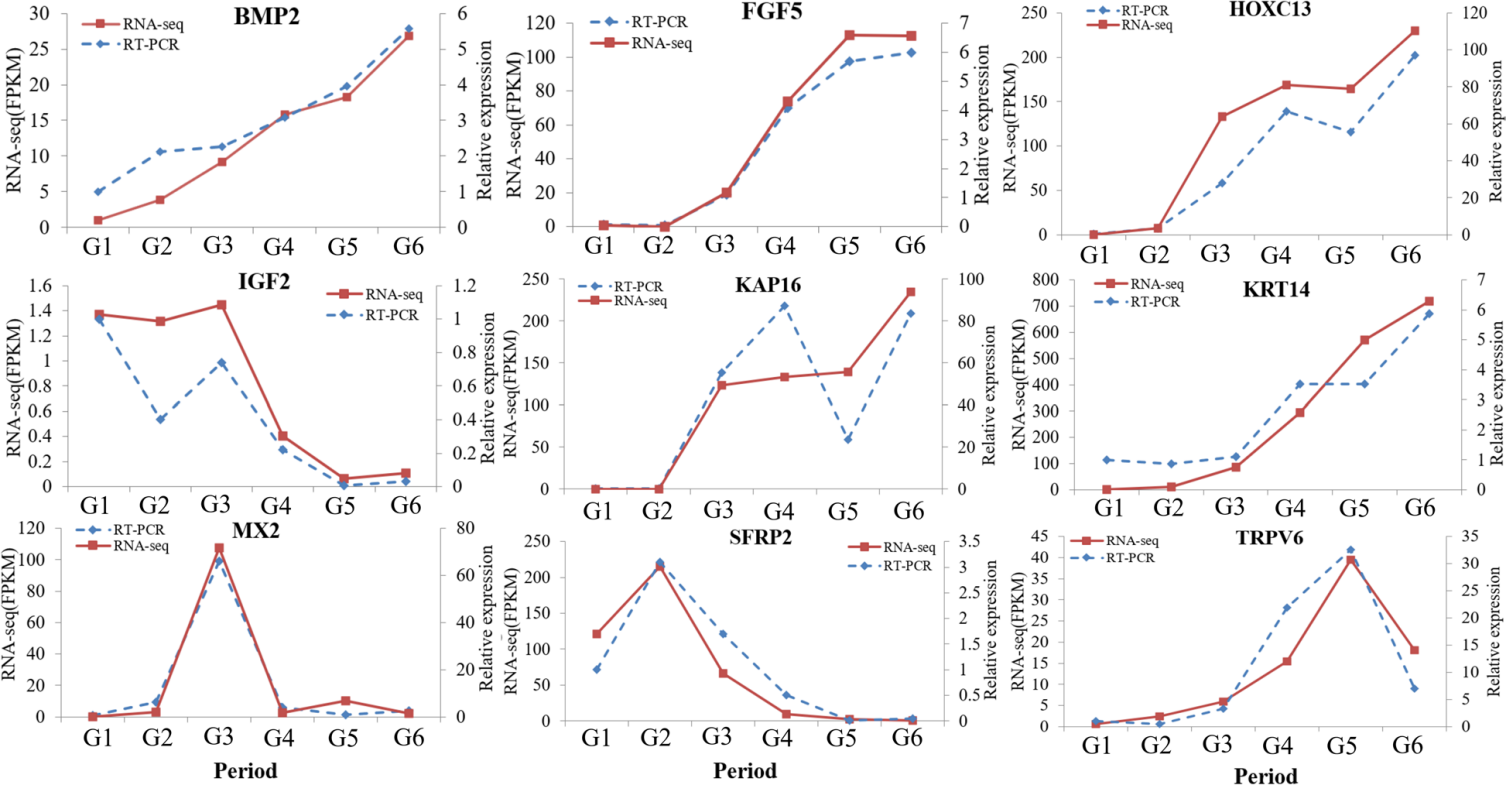
Results of RT–PCR and RNA-seq. The x-axis represents the time period, and the y-axis represents the relative expression

We compared the Real-time PCR (RT–PCR) results with the RNA-seq results (Fig. 8) and found that the expression levels of the *BMP2*, *FGF5*, *HOXC13*, *IGF2*, *KRT14*, *MX2*, *SFRP2*, *KAP16* and *TRPV6* genes in skin were consistent with the RNA-seq results. The results of *KAP16* gene expression from G3 to G5 were not consistent with the sequencing results, but the expression trends were consistent in other periods. Therefore, we concluded that the sequencing results were accurate and reliable.

## Discussion

The characterization of the molecular mechanisms controlling differentiation and proliferation in mammalian HFs is central to our understanding of the regulation of normal hair growth, the foundations of hereditary hair loss diseases, and the origins of follicle-based tumors. Zhao et al. [17] studied the morphogenesis of sheep HFs at six developmental stages using the hematoxylin and eosin (H&E) staining method and showed the asynchronous development of sheep HFs. The Pc and DC started to form at E65, indicating the induction of HFs. At E85, the number of PFs increased and the SFs started to form. At E105, the SFs started to differentiate, and the number of secondary-derived follicles increased at E135. At E135, hair follicles matured with a complete structure, and majority hair shafts emerged through the epidermis. At P7 and P30, HFs entered into the anagen phase, during which the root of the hair divides rapidly, adding to the hair shaft (HS) [17]. Results are consistent with those reported by Rogers [14]. Liu et al. [18] studied the differential expression of genes during HF growth and development in Aohan fine wool sheep and concluded that 83 genes were differentially expressed. Nai [19] assembled and analyzed transcriptome data from cashmere goat skin and HFs and obtained 617 DEGs in primary and secondary HFs. Liu [20] found 4581 DEGs among HF development-related genes in inner Mongolian cashmere goats. Gao [21] f found a total of 2059 DEGs at the E60, E120, and newborn (NB) stages. In our study, a total of 7879 DEGs were identified in the six HF development stages of SBM sheep. Compared with previous studies, the current study identified more DEGs. The number of novel DEGs identified in this study was 12623. There were significantly more novel DEGs than known DEGs, probably because the test samples were newly obtained from the SBM variety and because this study is the first to investigate the development of HFs in SBM sheep. Although there have been many studies on HF development-related genes, it is necessary to conduct in-depth research on their functions [22].

In the K-means analysis and WGCNA, we found several DEGs, such as *FZD1* [23, 24], *GLI2*, *KRT25* [25, 26], *LAMA5* [27, 28], *LRP4* [29, 30], *SOSTDC1* [31, 32], *TGFβ2* [33, 34], *TMEM79* [35], *BMP7* [36, 37], *WNT10A* [38], *ZDHHC21* [39], *SOX10* [40, 41], *ITGB4* [42], *KRT14* [43], and *ITGA6* [44], which are associated with the development of the epidermis and HF. They are also related to the development of the epithelium. Functional enrichment analysis showed that the DEGs were significantly enriched in negative regulation of the canonical Wnt signaling pathway, HF development, negative regulation of the BMP signaling pathway, establishment of the skin barrier, and positive regulation of epidermal cell differentiation and skin development, highlighting the central roles of these DEGs in hair morphogenesis. Notably, the fate of HFs is affected by typical Wnt/β-catenin signaling, BMP signaling, the TGF-β signaling pathway, the PI3K-Akt signaling pathway, and the Hippo signaling pathway.

**Fig.9:**
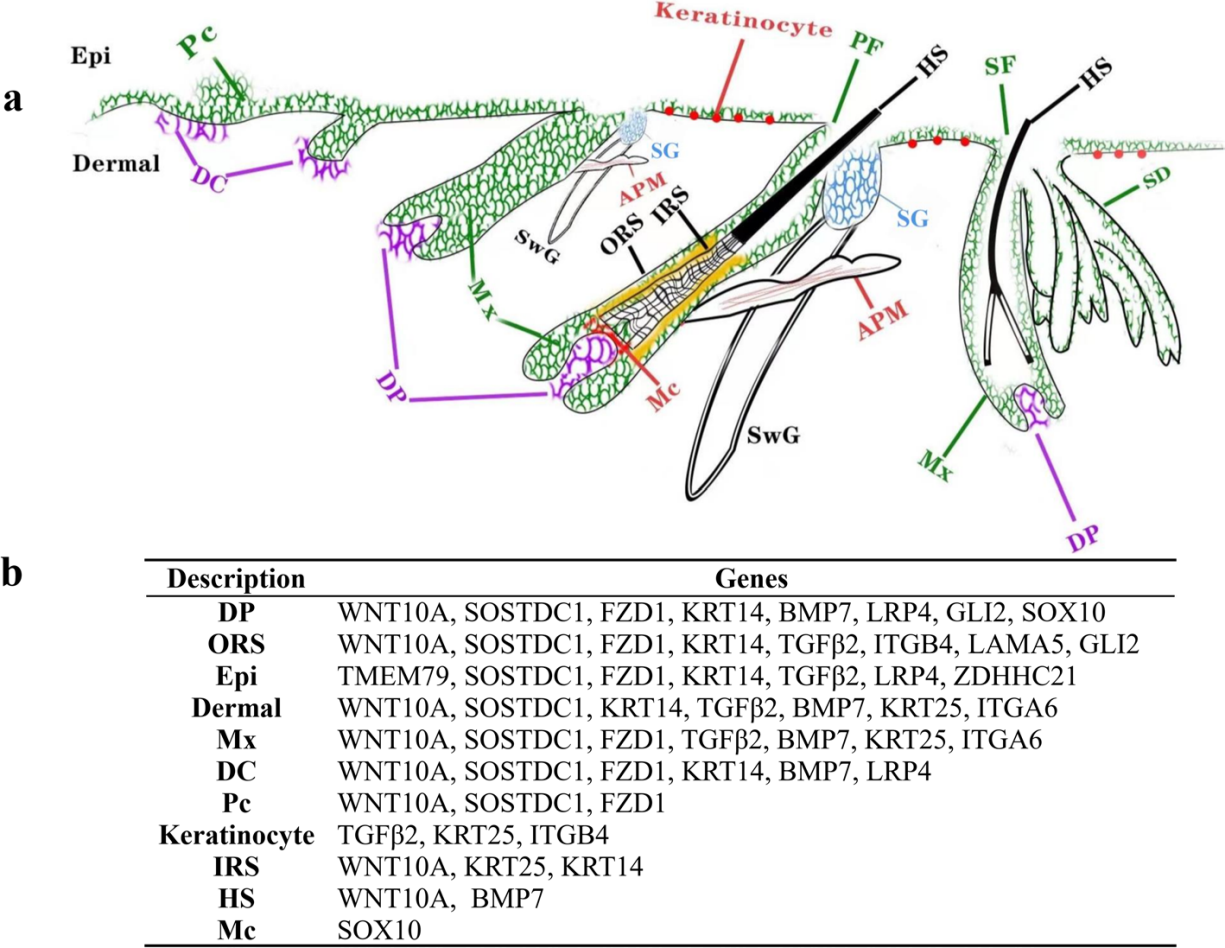
**a** Diagrams of HF development in Merino sheep. **b** Correspondence between different cell types and DEGs. *Epi* epidermal, *Pc* placode, *DC *dermal condensate, *DP* dermal papilla, *Mx* matrix, *SG* sebaceous glands, *SwG* Sweat gland, *AMP* Arrector pili muscle, *Mc* melanocytes, *ORS* outer root sheath, *IRS* inner root sheath, *PF* primary follicle, *SF* secondary follicle, *SD* secondary-derived follicle,* HS *hair shaft

Embryonic HF development and postnatal hair growth rely on intercellular communication within the epithelium and between epithelial and mesenchymal cells [19]. Rendl et al. [45] takes a novel multicolor labeling approach, using cell type–specific transgenic expression of red and green fluorescent proteins in combination with immunolabeling of specific antigens, to isolate pure populations of dermal papilla (DP) and four of its surrounding cell types: dermal fibroblast (DF), melanocytes (Mc), matrix (Mx) and outer root sheath (ORS). They found there was a remarkably high correlation between the DP and DF, and the Mx and ORS, respectively, highlighting the common mesenchymal origin of DP and DF and the close lineage relationship of Mx and ORS. The lowest correlation occurred between DP and Mx, revealing striking differences between the two populations whose signaling exchange orchestrates the dynamics of the hair growth. In recent years, with the development of single-cell sequencing technology, the classification of cells related to HF development and the analysis of differential genes have been studied [46–48]. Comparing the results of previous studies with the results of this study, we found the role of some genes in different HF cell populations (Fig. 9). These genes included the DP signature genes (i.e. *WNT10A*, *SOSTDC*, *BMP*, *KRT14*,* FZD1*, *GLI2*, *LRP4*, and *SOX10*), the ORS signature genes (i.e. *LAMA5*, *WNT10A*, *SOSTDC1*,* TGFβ2*, *ITGB4*, *KRT14*, *FZD1*, *GLI2*), the inner root sheath (IRS) signature genes (i.e. *WNT10A*, *KRT14*), the Mx signature genes (i.e. *KRT25*, *SOSTDC1*, *ITGA6*, *BMP7*, *FZD1*, *WNT10A*), the epidermal signature genes (i.e. *TMEM79*, *KRT14*, *SOSTDC1*, *LRP4*, *TGFβ2*, *FZD1*, *ZDHHC21*), the dermal signature genes (i.e. *SOSTDC1*, *KRT14*, *KRT25*, *TGFβ2*, *BMP*, *WNT10A*, *ITGA6*), the DC signature genes (i.e. *WNT10A*, *SOSTDC1*,* BMP7*, *KRT14*, *FZD1*, *TGFβ2*, *LRP4*), the Pc signature genes (i.e. *WNT10*A, *SOSTDC1*, *FZD1*), the keratinocyte signature genes (i.e. *TGFβ2*, *ITGB4*, *KRT14*). In addition, the hair disorder– associated genes (i.e. Gli2). The DP and ORS cells of the HF express Gli1 and Gli2 during anagen [49]. Närhi, et al. [32] found that Sostdc1 limits the number of developing HFs, and the size of primary hair placodes. Sostdc1 is essential for suppression of hair follicle fate in the normally hairless nipple epidermis. Suggest that functions of Sostdc1 can be largely attributed to its ability to attenuate Wnt/β-catenin signaling. Cross-talk of BMP and Wnt signaling pathways has been implicated in many aspects of biological events during embryogenesis and in adulthood. A secreted protein Wise and its orthologs (Sostdc1) have been shown to modulate Wnt signaling and also inhibit BMP signals and Wise also binds LRP4, a member of the LRP family functioning inhibitory to Wnt signals [50]. A homozygous missense mutation within KRT25 that causes autosomal recessive woolly hair in humans, which is consistent with findings in mutant mice. The identification of a KRT25 mutation as a cause of woolly hair in humans [51, 52]. All-trans-retinoic acid could inhibit hair follicle growth via inhibiting proliferation and inducing apoptosis of DPCs partially through the TGFβ2/Smad2/3 pathway [33]. Reddy et al. [24] found that expression of FZD1 in the placode correlates with expression of WNT10A and WNT10B in the placode. They suggest that canonical WNT signaling is likely activated in the placodes and DC of developing hair follicles by WNT10A and WNT10B, expressed in and secreted from epithelial cells, binding to FZD1, expressed in the placode epithelium and DC. Expression of FZD1 is detected in the DP and Mx of anagen follicles, and could potentially interact with DP and Mx cells, in particular WNT10A. In postnatal HFs in full anagen, FZD1 express in the ORS. At the germ and bulbous peg stages, WNT10A and WNT10B are expressed continuously in follicular epithelium during these later stages of morphogenesis. At the bulbous peg stage, WNT10A and WNT10B are strongly expressed in a cone of epithelial cells surrounding the DP. In rat whisker HFs WNT10A was expressed in the ORS, IRS, Mx and HS of anagen follicles [53].

The characteristics of HF development and postpartum regeneration are significantly altered in microanatomy and cell viability experiments. HF development is controlled by a variety of signaling pathways, transcription factors and epigenetic regulatory factors (including miRNAs) [54]. Some studies based on HF gene regulatory networks have shown that the Wnt [10], TGF-β [55, 56], MAPK [57], Shh [58], BMP [59], PI3K-Akt [60, 61], Notch [62] and JAK-STAT [63] signaling pathways are widely involved in HF development, morphogenesis and circulation. The Wnt signaling pathway is capable of stimulating the conversion of HF stem cells from a resting state to a growing state. In most cases [64], the MAPK signaling pathway plays a major role, while the TGF-β signaling pathway [65] is the lead activator of other signaling pathways, and the Shh, Notch and JAK-STAT signaling pathways play fine-tuning roles [66].

In our study, we found that the PPAR signaling pathway was significantly enriched in G3/G2 and G4/G3, indicating that it plays an important role in the development stage of secondary HFs and secondary acquired HFs. Recent studies have shown that HF morphogenesis is not only related to HF cell proliferation and differentiation but also affected by other cells around the HF, such as sebaceous glands and sweat glands [67–69]. PPARs are members of the nuclear hormone receptor family and have become recognized as important mediators of lipid metabolism in adipocytes and sebaceous glands [70, 71]. A group of in vitro studies have demonstrated that PPARs play significant roles in cell differentiation, lipid synthesis and fatty acid uptake [65]. In addition, PPARs are engaged in the regulation of keratinocyte differentiation and the formation of functional skin barriers [72, 73].

Here, by analyzing DEGs at specific stages of development, we have provided insight into the genetic structure behind the complex biological process of HF development. However, functional verification experiments are needed to validate the candidate genes for traits related to HF development identified in this study and to evaluate their effectiveness in animal breeding. Previously, we performed sequencing to analyze noncoding RNAs [1, 17] and methylation [74]. In future research, the sequencing of skin should be performed using single-cell RNA-seq to determine the molecular drivers of HF development. In general, the discovery of candidate genes related to sheep HF development will help us improve and utilize sheep breeds. Finally, providing the necessary animal products for human beings is the ultimate goal of our animal husbandry workers.

## Conclusion

In summary, we used RNA-seq to sequence the mRNAs in the six developmental stages of SBM HF and conducted bioinformatics analysis. We identified a total of 7879 DEGs and 12623 novel DEGs and determined 15 key candidate genes that are significantly related to HF morphogenesis. The DEGs selected in this study and the associated pathways (the TGF-β, Wnt, Hippo, PI3K-Akt, MAPK, PPAR and Hedgehog signaling pathways) are involved in HF development. Our study provides a new reference for the molecular basis of HF development and lays a foundation for further improving sheep HF breeding. Candidate genes related to HF development were found, which provided a theoretical basis for molecular breeding for the production of fine wool sheep. These results are a valuable resource for biological investigations of fleece evolution in animals and supply potential clues for understanding the molecular mechanisms of human hair development.

## Materials

### Animal selection and skin tissue collection

SBM is a subtype of the Merino breed of sheep in China and is famous for its excellent wool quality and yield and high survival rate. The tested individuals were selected from the sheep herd located at the Xinjiang Kechuang Animal Husbandry Breeding Center, Urumqi County (Latitude 43°01′08"–44°06′11"N, Longitude 86°37′56"–88°58′22"E), Xinjiang, China. Eighteen healthy SBM ewes (2–3 years old; mean fiber diameter (MFD), 18.1 ± 0.5 μm) were artificially inseminated with fresh sperm from an SBM ram (3 years old; MFD, 19.0 ± 0.4 μm) and then managed in the same flock. The day of insemination was designated embryonic day 0 (E0). Embryos were collected from 12 pregnant ewes (by electrocution followed by exsanguination) at four embryonic days (i.e., E65, E85, E105, and E135). Skin tissues were collected immediately after euthanasia. The skin tissues of postnatal lambs were collected in vivo at a depth of approximately 2 cm^2^ × 3 mm at P7 and P30. G1 to G6 represent hair follicles developmental at six stages (i.e. E65 to P30). All eighteen skin tissues were collected from the right mid-side region behind the shoulder blade of each individual and rinsed in 1 × phosphate-buffered saline (PBS). The samples were cut into small pieces, quickly placed into liquid nitrogen, and subsequently stored at − 80 °C for RNA-seq.

### Total RNA isolation, library construction, and sequencing

Total RNA was isolated from the tissues using TRIzol reagent (Invitrogen, Carlsbad, CA, USA). The RNA quality was verified using a 2100 Bioanalyzer RNA Nano Chip (Agilent Technologies, Santa Clara, CA, USA), and the total RNA content was measured using a NanoDrop ND-2000 Spectrophotometer (NanoDrop Technologies, Wilmington, DE, USA). Only samples with RNA integrity number (RIN) scores > 8 were used for sequencing. Eighteen cDNA libraries were constructed using an NEBNext® Ultra™ Directional RNA Library Prep Kit for Illumina (NEB, USA) according to the manufacturer’s instructions, after which rRNA was removed using an Epicenter Ribo-Zero rRNA Removal Kit (Epicenter, USA). The purified libraries were quantified using a Qubit® 2.0 Fluorometer (Life Technologies, USA) and validated using an Agilent 2100 Bioanalyzer (Agilent Technologies, USA) to confirm the insert size and calculate the molar concentration. Sequencing was performed using an Illumina HiSeq 2000 Genome Analyzer (Illumina Inc., San Diego, CA, USA).

### Differential expression analysis and clustering of mRNAs

For known genes, we used Cuffdiff (v2.1.1) to calculate fragments per kilobyte per million reads (FPKM) for mRNAs in each sample based on TopHat BAM files and reference GTF files [75]. These counts were used to analyze differential gene expression using edgeR software [76]. For novel genes, we put together the genomic mapping results from all sequenced reads. Sequencing reads were assembled using Cufflinks (version 2.2.1) and compared with the known sheep genome (Ensemble Oar _v 3.1) using Cuffcompare to discover novel genes (relative to the original gene annotation file). Finally, HTSeq was used to quantify the expression of Novel genes. DEGs and Novel DEGs that were differentially expressed between any two comparison groups of samples representing specific developmental stages were identified using edgeR. The threshold value of *P*-value [77, 78] is determined by controlling FDR (false discovery rate), and the corrected *P*-value is Q-value. At the same time, we calculated the differential expression multiple (fold change) according to the FPKM value. The screening conditions of differential genes are as follows: Q-value ≤ 0.05 and fold change ≥ 2 were considered differentially expressed between the adjacent comparison groups (comparisons: G2/G1, G3/G2 G4/G3, G5/G4, G6/G5, and G6/G1) [79]. The expression patterns of DEGs were analyzed by systematic clustering to explore the similarities and relationships between the different libraries. Furthermore, the DEGs were subjected to K-means clustering using the Euclidean distance method associated with complete linkage on the BMK Cloud platform (https://www.biocloud.net/).

### Functional enrichment analysis and gene annotation

Functional annotation was performed using the GO (http://geneontology.org) and KEGG (http://www.genome.ad.jp/kegg/) databases [80] based on GO and KEGG pathways. These analyses were conducted for the DEGs identified in each tissue at each stage of HF development, which were significantly enriched in dynamic expression patterns. The BPs and metabolic pathways significantly associated with the gene lists were determined based on their FDR [81]. Functional evidence was obtained on the basis of the relationships between the significant GO terms (FDR < 0.05) and the DEGs [82]. The Database for Annotation Visualization and Integrated Discovery (DAVID) 6.8 (https://david.ncifcrf.gov/tools.jsp) was used to perform functional annotation of the genes that were significantly enriched in the expression patterns [83].

### Construction of a PPI network

Following the integration of the protein information in the Search Tool for the Retrieval of Interacting Genes/Proteins (STRING) (https://string-db.org/) database with the DEGs, a PPI network of the identified DEGs was established. PPI network analysis was performed using STRING and Cytoscape software V3.8.0 (http://www.cytoscape.org/). First, STRING was utilized to analyze the correlation coefficients between genes. Interacting pairs with confidence scores > 0.5 were selected to build the PPI network, and the Matthews correlation coefficient (MCC) algorithm was used to calculate hub genes and the selected top 120 genes were visualized using Cytoscape software.

### K-means analysis and WGCNA

DEGs were clustered with the R package K-means function, where K = 10 within the cluster package according to the Euclidean distance. WGCNA [84, 85] was applied to the FPKM expression data. A coexpression network was constructed with a beta value of 4. We calculated the coefficients of gene dissimilarity, performed hierarchical clustering of the genes and then determined the gene modules by the dynamic tree cut method. Through clustering analysis, modules close to each other were merged into new modules. Before WGCNA, we identified and filtered the selected gene set. We removed low-quality genes with an unstable impact on the results to improve the accuracy of network construction. The filtering criteria in this study were as follows: for each gene, the maximum count value in all samples was > 50, and the count was > 20 in at least 16 samples. The modules were functionally annotated using DAVID. Highly connected genes in each module, which are also known as hub genes, may play important roles in the module. Hub genes are conserved to a certain extent and are at the core of the gene coexpression network. These genes can act as genetic buffers to reduce the impacts of other gene mutations. We identified the top 150 hub genes in the modules that were most closely related to HF development differences, that is, the 150 genes with the highest connectivity in the modules, and used Cytoscape software to map the gene–gene interaction network to visualize the gene relationships.

### Validation of RNA-seq data

Several differentially expressed mRNAs involved in HF development were selected and confirmed by RT–PCR with *GAPDH* as an internal reference. The primers used for RT–PCR are listed in Additional file 11: Table S7. Total RNA from the samples used for high-throughput RNA-seq was isolated and converted into cDNA using a PrimeScript™ RT Reagent Kit with gDNA Eraser (TaKaRa, Japan). RT–PCR was carried out on a CFX96™ Real-Time System (Bio–Rad, USA) using a TB Green Premix Ex Taq™ kit (TaKaRa, Japan) according to the manufacturer’s instructions. The thermal cycling conditions used in RT–PCR were 95 °C for 30 s followed by 40 cycles of 95 °C for 5 s and 60 °C for 30 s. A reaction volume of 20 μL was used for RT–PCR according to the manufacturer’s protocol. The specificity of the SYBR Green PCR signal was confirmed by melting curve analysis. The RT–PCR experiments were performed in triplicate, and the average Ct value was used for further analysis. The 2^−ΔΔCt^ method was used to determine the relative mRNA abundance.

## Supplementary Information

**Additional file 1:** **Table S1.** Result Statistics of mRNA Genome Comparison. Mapping ratio=Mapped reads/All reads, Mapped Unique reads: reads that have only one position in the genome.

**Additional file 2:** **Fig. S1.** Heatmap of DEGs during the hair follicle morphogenesis. The x-axis represents the sample number, where S1 to S3 is G1; S4 to S6 is G2; S7 to S9 is G3; S10 to S12 is G4; S13 to S15 is G5; S16 to S18 is G6.

**Additional file 3:** **Fig. S2.** Enrichment analysis of all DEGs. (a)Top 30 of GO enrichment; (b)Top 30 of pathway enrichment.

**Additional file 4:** **Table S2.** GO enrichment of DEGs.

**Additional file 5:** **Table S3.** KEGG pathway of DEGs.

**Additional file 6: Fig. S3.** Go and KEGG network diagram during the hair follicle morphogenesis. (a-f) represents the comparison group of G1-G6. Circle is represented gene, square is represented KEGG; triangle is represented GO term.

**Additional file 7: Fig. S4.** K-means clustering analysis of differentially expressed mRNAs among the six comparison groups.

**Additional file 8:** **Table S4.** GO analysis of K-means clusters.

**Additional file 9:** **Table S5.** GO analysis of module in WGCNA.

**Additional file 10:** **Table S6.** KEGG pathway of module in WGCNA.

**Additional file 11:** **Table S7.** Sequence of primers.

## Data Availability

All RNA-seq data generated in this study were submitted to the NCBI SRA database under BioProject No. PRJNA705554.

## Abbreviations

HF: Hair follicle
DEGs: Differentially expressed genes
SBM: Subo merino
PF: Primary follicle
SF: Secondary follicle
SD: Secondary-derived follicle
Pc: Placode; DC: Dermal condensate
DP: Dermal papilla
SG: Sebaceous gland
ORS: Outer root sheath
IRS: Inner root sheath
DF: Dermal fibroblast
Mx: Matrix
Mc: Melanocytes
Epi: Epidermal
SwG: Sweat gland
HS: Hair shaft
AMP: Arrector pili muscle
BMP: Bone morphogenetic protein
TGF-β: Transforming growth factor beta
GO: Gene ontology
KEGG: Kyoto Encyclopedia of Genes and Genomes
BP: Biological processes
CAM: Cell adhesion molecule
HCM: Hypertrophic cardiomyopathy
ECM: Extracellular matrix
PPAR: Peroxisome proliferator-activated receptor
MAPK: Mitogen-activated protein kinase
mRNA: Messenger RNA
NB: Newborn
PBS: Phosphate-buffered saline
RIN: RNA integrity number
FPKM: Fragments per kilobase of exon model per million mapped fragments
FC: Fold change
FDR: False discovery rates
DAVID: Database for Annotation, Visualization, and Integrated Discovery
WGCNA: Weighted gene co-expression network analysis
PPI: Protein–protein interaction
RT–PCR: Real-time PCR
STRING: Search Tool for the Retrieval of Interacting Genes/Proteins
