# Supplementary material for: Gene network analysis reveals candidate genes related with the hair follicle development in sheep
Source: BMC Genomics. 2022 Jun 8;23:428. doi: 10.1186/s12864-022-08552-2 (PMC9175362; doi:10.1186/s12864-022-08552-2)
Supplement: Supplementary file 2 — Additional file 2: Fig. S1. Heatmap of DEGs during the hair follicle morphogenesis. The x-axis represents the sample number, where S1 to S3 is G1; S4 to S6 is G2; S7 to S9 is G3; S10 to S12 is G4; S13 to S15 is G5; S16 to S18 is G6. [file 12864_2022_8552_MOESM2_ESM.docx]

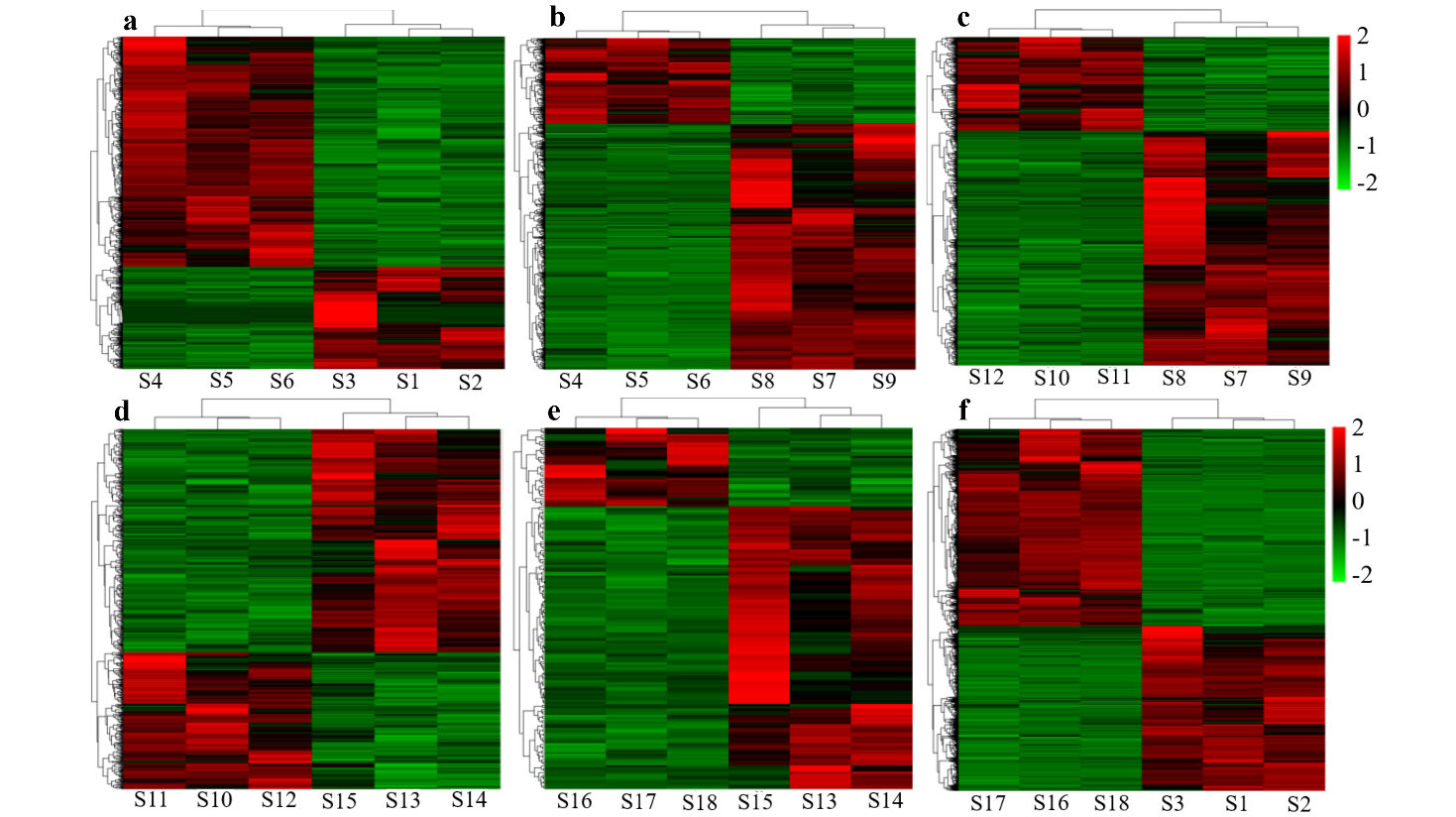


Fig.S1. Heatmap of DEGs during the hair follicle morphogenesis. The x-axis represents the sample number, where S1 to S3 is G1; S4 to S6 is G2; S7 to S9 is G3; S10 to S12 is G4; S13 to S15 is G5; S16 to S18 is G6.
