# Supplementary figures and images for: Gene network analysis reveals candidate genes related with the hair follicle development in sheep
Source: BMC Genomics. 2022 Jun 8;23:428. doi: 10.1186/s12864-022-08552-2 (PMC9175362; doi:10.1186/s12864-022-08552-2)

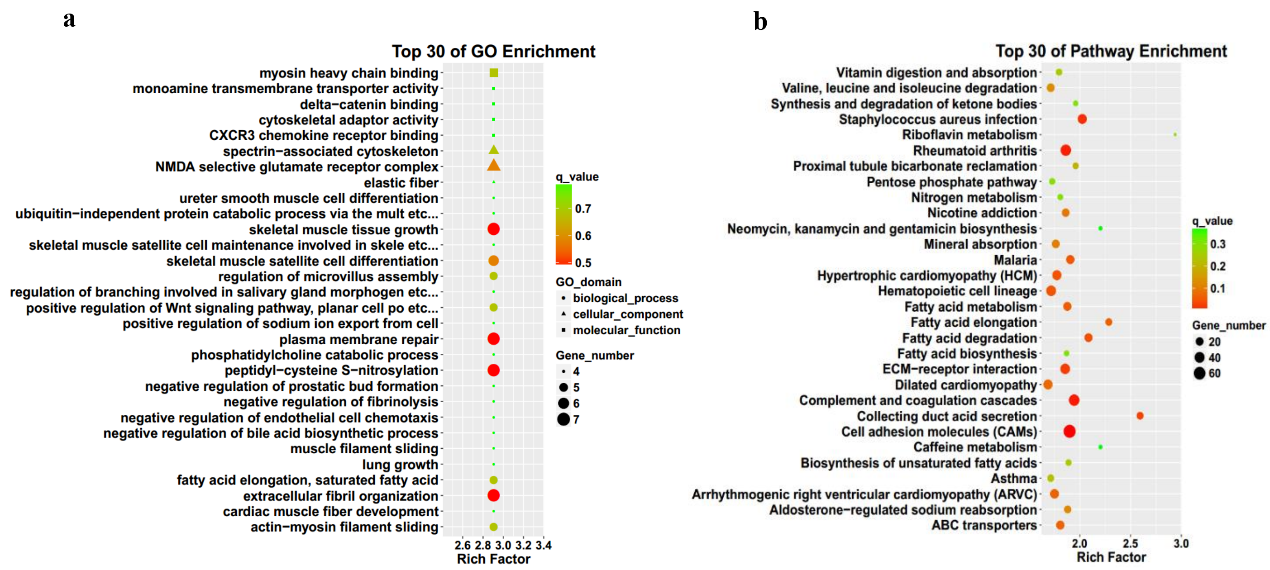


Fig.S2. Enrichment analysis of all DEGs. (a)Top 30 of GO enrichment; (b)Top 30 of pathway enrichment.

Supplement: Supplementary file 3 — Additional file 3: Fig. S2. Enrichment analysis of all DEGs. (a)Top 30 of GO enrichment; (b)Top 30 of pathway enrichment. [file 12864_2022_8552_MOESM3_ESM.docx]
