# Supplementary material for: Gene network analysis reveals candidate genes related with the hair follicle development in sheep
Source: BMC Genomics. 2022 Jun 8;23:428. doi: 10.1186/s12864-022-08552-2 (PMC9175362; doi:10.1186/s12864-022-08552-2)
Supplement: Supplementary file 6 — Additional file 6: Fig. S3. Go and KEGG network diagram during the hair follicle morphogenesis. (a-f) represents the comparison group of G1-G6. Circle is represented gene, square is represented KEGG; triangle is represented GO term. [file 12864_2022_8552_MOESM6_ESM.docx]

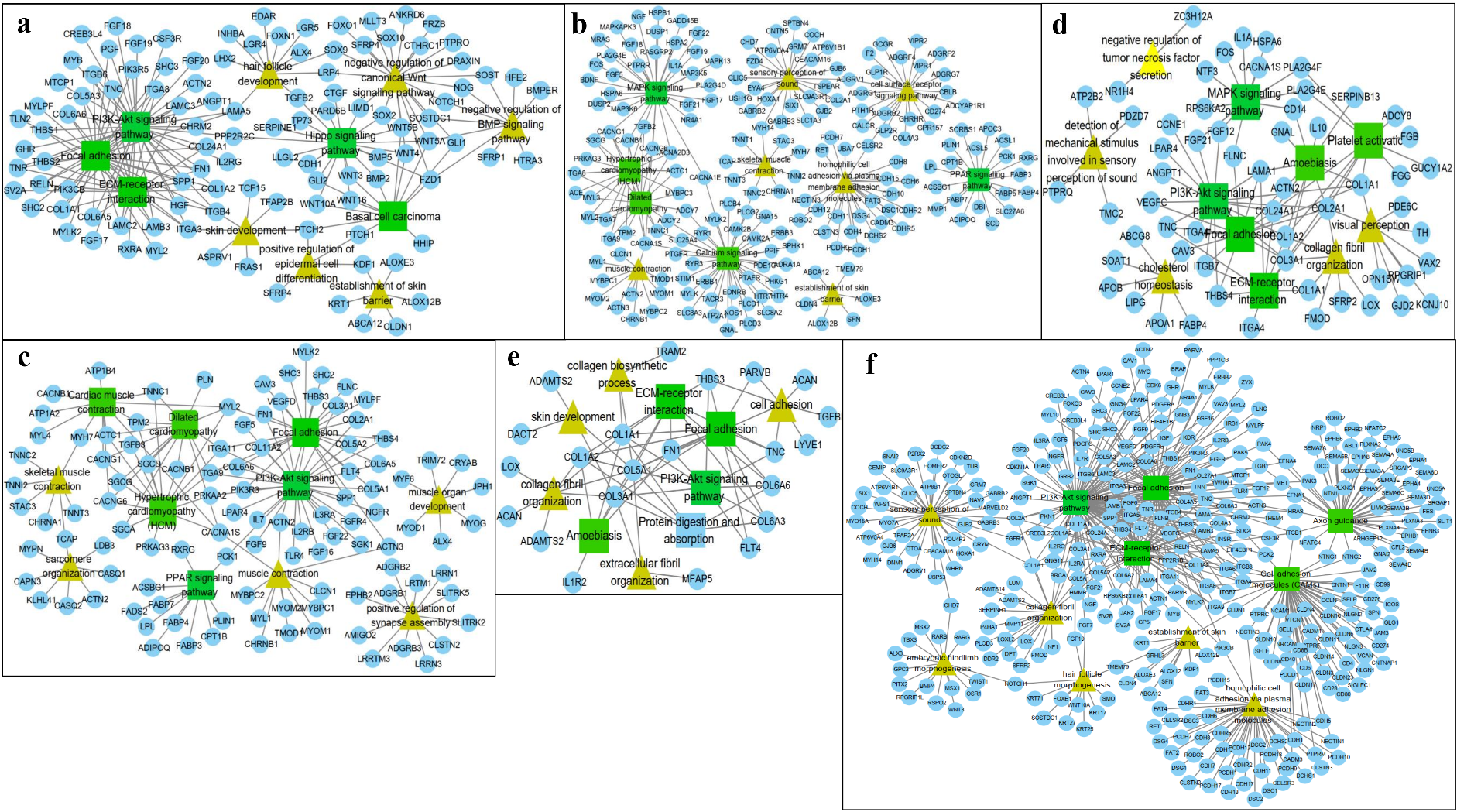


Fig. S3. Go and KEGG network diagram during the hair follicle morphogenesis. (a-f) represents the comparison group of G1-G6. Circle is represented gene, square is represented KEGG; triangle is represented GO term.
