# Supplementary material for: Gene network analysis reveals candidate genes related with the hair follicle development in sheep
Source: BMC Genomics. 2022 Jun 8;23:428. doi: 10.1186/s12864-022-08552-2 (PMC9175362; doi:10.1186/s12864-022-08552-2)
Supplement: Supplementary file 7 — Additional file 7: Fig. S4. K-means clustering analysis of differentially expressed mRNAs among the six comparison groups. [file 12864_2022_8552_MOESM7_ESM.docx]

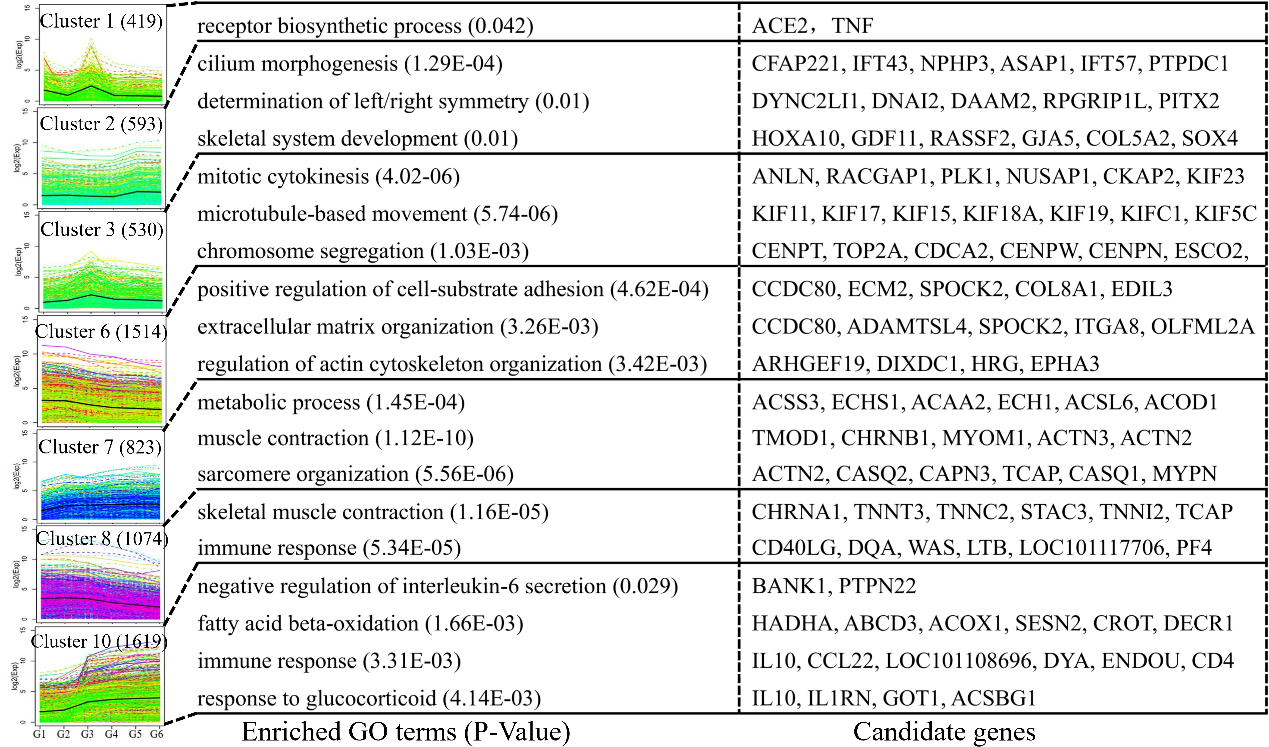


Fig. S4. K-means clustering analysis of differentially expressed mRNAs among the six comparison groups.
